# Effects of rejection intensity and rejection sensitivity on social approach behavior in women

**DOI:** 10.1371/journal.pone.0227799

**Published:** 2020-01-17

**Authors:** Violetta K. Schaan, André Schulz, Michael Bernstein, Hartmut Schächinger, Claus Vögele

**Affiliations:** 1 Institute for Health and Behaviour, Department of Behavioural and Cognitive Sciences, University of Luxembourg, Esch-sur-Alzette, Grand-Duchy of Luxembourg; 2 Department of Psychology and Social Sciences, Penn State Abington, Abington, Pennsylvania, United States of America; 3 Department of Biological and Clinical Psychology, University of Trier, Trier, Germany; University of Rome, ITALY

## Abstract

**Objective:**

Perceived rejection plays an important role for mental health and social integration. This study investigated the impact of rejection intensity and rejection sensitivity on social approach behavior.

**Method:**

121 female participants were randomly assigned to one of three conditions differing in the degree of induced rejection (inclusion, medium rejection, severe rejection). Thereafter they were asked to interact with an unknown person during a touch-based cooperative task.

**Results:**

Participants high in rejection sensitivity sought significantly less physical contact than participants low in rejection sensitivity. Individuals in the medium rejection condition touched their partners more often than those in the included condition, while no difference between included and severely rejected participants could be observed.

**Conclusions:**

The results suggest that the intensity of rejection matters with regard to coping. While participants in the medium intensity rejection condition aimed to ‘repair’ their social self by seeking increased contact with others, severely rejected participants did not adapt their behavior compared to included participants. Implications for therapy are discussed.

## Introduction

Humans have a strong need to belong and to connect to others [1]. Social belonging and social support are crucial for psychological well-being and physical health [2,3]. The initiation of social relationships, however, requires social skills, and social relationships must evolve from an initial getting-acquainted stage to meaningful and stable social bonds over time [4,5]. This natural course of the development of social bonds is mastered successfully by some individuals while others struggle already at the initiation level. Fears of being rejected for instance might play a crucial role in how social encounters are initiated and kept. Also, recent rejections might affect future interactions with strangers. Both, an individual’s trait fear of rejection as well as a previous experience of rejection of different intensities might interact with one another and influence how social encounters are initialized in the future. This study aims to investigate the impact of rejection on behavior and it is therefore important to control for trait and state-like features to discern whether the observed effects are a result of the trait feeling of being rejected or the quality of the situation. This is the first study offering an approach of combining both factors together allowing us to report state effects that are independent of trait perceptions.

### Threats to social relationships

Threats to social relationships, such as rejection from peers, can have serious consequences. Lack of attachment is linked to a range of ill effects on health, adjustment, and well-being [6–8]. Social isolation and loneliness have, for instance, been shown to result in cardiovascular changes [6], poor sleep [9], impaired immunity [10], depression [11,12] and increased mortality [13]. Research on the consequences of ostracism shows that it negatively impacts cognitive [14] and self-regulatory abilities [15], as well as increasing the probability for aggressive behavior [16]. In contrast to these apparent maladaptive effects, ostracism also results in increased attention to social cues [17–20], better memory for social information [21], reductions in stereotyping [22] and increased helping behavior [23]. Perception of threats to one’s social belonging can, for example, be related to the current situation (e.g. no one shows up to your birthday party), or constitute a personality disposition (e.g., rejection sensitivity (RS)) regardless of the current social situation. Independent of the origin of rejection, they can lead to severe emotional (and even physical) pain and stress to the person concerned [24,25]. In this paper, we did not distinguish between ostracism, exclusion, and rejection. There are certainly differences between these concepts, with Williams [26], for example, arguing that ostracism is the experience of being ignored or rejected while exclusion requires less active behavior on the part of the perpetrators. There is also some evidence concerning the differences between such experiences (see Molden and colleagues [27] for an examination of the difference between being ignored and being rejected). The vast majority of work in this area, however, has not considered how these experiences differ or if they do at all. While we see this as a potentially interesting area of future work, we think it is beyond the scope of the current paper.

### Intensity of social rejection

Some have suggested that the severity of a social rejection experience plays a crucial role in how others respond to rejection [28]. Medium intensity social stress (anticipating losing some but keeping other friends) has been shown to increase pain sensitivity, whereas severe social stress (anticipating losing all relevant social ties) was related to increases in pain tolerance [28]. Severe rejection may thus lead to a numbing response (both emotionally and physically) [24].

Physical pain is a motivator to engage in behaviors that alleviate the pain, e.g. taking pain medications or resting behaviors. In the present study, we, therefore, expect that social pain motivates similar behavior (e.g. re-affiliation or social retreat) to alleviate the social pain. It is anticipated that severe social injury initiates social retreat, in line with the pain overlap theory [24] and work by DeWall and Baumeister [29] showing that people become emotionally and physically numb and show less interest in being around others, when being severely rejected. Retreat should, however, only be observable for severely rejected participants as moderate rejection results in hypersensitivity to pain and an increased emotional pain response, which–according to Bernstein and Claypool [28]—should facilitate adaptive responding, i.e. re-affiliation. We, therefore, expect medium severity rejection to motivate participants to seek social contact, whereas high severity rejection should result in people retreating from social situations.

These theoretical assumptions are in line with the threat and challenge model by Blascovich [30], as stress/rejection of a medium intensity may be experienced as challenging, allowing oneself to experience the negative affect associated with rejection and promoting the motivation to reduce this negative affect by re-affiliation in the future (e.g. motivation to enlarge one’s social network again after loss). Severe threat to the social self (as provoked by severe rejection intensity) might overwhelm the individual, resulting in emotional and physical numbing and withdrawal, with the aim to protect oneself from the terrifying pain of the experienced rejection and leading to a passive social attitude, promoting social isolation [28,30,31].

### Challenge and threat as response to rejection

Stress reactions have been widely investigated with a focus on task performance, demonstrating that the degree with which stress intensity impacts on task performance greatly depends on the way the person faced with this situation interprets it. While threat—characterized by the feeling that resources are missing for successful task completion—has been shown to impede performance, challenge—characterized by the feeling that successful coping is possible with the resources at hand—is related to better test results [32–36]. Whether different intensities of social rejection also affect performance in social cooperative interactions, however, remains unclear, as the two goals (connecting vs. performing) might work against each other for instance due to a competition of limited attentional resources [37,38].

### Rejection sensitivity

Trait rejection sensitivity (RS) refers to a disposition to “anxiously expect, readily perceive and overreact to rejection” ([39], p. 1327) resulting in a lower sense of belonging and lower perceived control over social interactions [39–41]. RS has been demonstrated to be a significant predictor of mental health issues such as depressive symptoms, anger, aggression and interpersonal problems [39, 40, 42–44]. How RS influences social behavior might depend on situational cues: avoiding contact with others might forestall rejection and prevent uncomfortable closeness in situations in which social withdrawal is impossible [45,46]. In contrast, expectations of rejection can trigger negative schemas [47], which prompt affective and behavioral overreactions including attempts to ‘regain’ control over others by using inappropriate social interactions [39,46]. Accordingly, individuals high in RS report more dissatisfaction in their close relationships which is related to increased jealousy, hostility and reduced supportiveness [39]. RS has been found to predict self-reported loneliness and social avoidance [48]. There are no studies, however, using an experimental approach to investigate the association between RS and behavior during social encounters with strangers in a controlled environment. Increased vigilance towards rejection and the persistent fear of being socially excluded in those with increased RS could lead to reluctance to socialize with strangers as they might want to protect themselves from possible rejection, thereby unwillingly reducing their chances to deepen social encounters. It could be argued, therefore, for RS to be related to reduced intensity of social interactions. To the best of our knowledge, there is no research on the effects of RS on performance in social cooperative interactions. The current study, therefore, aimed to examine this relationship in an exploratory design. We expect that individuals high in RS, as compared to individuals low in RS, are more distracted by social interactions and anxiety about the interaction at the expense of attention to a cognitive task and task performance.

### Study aims

Given the important implications of RS and loneliness on mental health and social integration [39, 41, 46], it is important to better understand the mechanisms linking RS and social interaction behavior. The current study aimed at investigating this question further by using an experimentally controlled, non-threatening social cooperative task. We expect RS to be related to reduced contact seeking during a social cooperative interaction (Ia). We also investigated if RS affects performance during a social interaction (Ib). In addition, we investigated possible effects of stress intensity on re-affiliative behaviors.

In view of the findings by Bernstein and Claypool [28], it is also important to investigate if rejection intensity selectively affects post-rejection social behavior. We expect medium intensity social stress to trigger more intense negative emotions (II) and more re-affiliative behavior (III), while no such increase in negative emotions (II) and social interactions (III) are expected for individuals under conditions of severe social stress [28]. Finally, we investigated whether experimentally induced rejection affects performance during a subsequent cooperative interaction task (IV). In this study we focused on female participants only to increase testing power by avoiding interactions by sex.

## Method

### Participants

The study was approved by the Ethics Review Panel of the University of Luxembourg (ERP-15-013 Reject LB/vg). German-speaking participants were recruited online via social networks, through university postings and university circular emails. Exclusion criteria were current medication, alcohol consumption >30 g/day, illicit drug intake within the last 3 months and current mental disorders that might influence the experimental results (e.g. depression, anxiety disorder, psychosis, suicidal ideations). One hundred-twenty one women participated in the study. Mean age was 23 years (SD = 4.55). Before the experimental session, participants were assessed using the structured clinical interview for DSM-IV major mental disorders and personality disorders. Forty out of 121 participants fulfilled the criteria for a major mental disorder, with 9 participants fulfilling the criteria for a personality disorder. Mean rejection sensitivity scores did not differ between participants who were diagnosed with a major mental or personality disorder and those without a diagnosis of a mental disorder (p>.05). Most participants came originally from Germany (N = 67) and Luxembourg (N = 43). Four participants identified as French, 1 as Italian, 1 as from Belgium, 1 from Brazil, 1 from Austria, 1 from Portugal, and 1 from Romania. One participant did not indicate her nationality. Educational background of the participants was high with 120 having a university entrance diploma, and one was about to finish grammar school. We conducted an a priori power calculation for F-test statistics using G*Power 3.1 [49]. The observations of Bernstein & Claypool [2. study; 28] with regard to pain sensitivity revealed an effect of η^2^ = .29, which stipulates a large effect based on Cohen’s conventions. As the main dependent variable was length of time of physical touch, we assumed a medium effect size (*f* = .30, 1-*β* = .80, N = 111). To overcome the effects of possible drop-outs, we over-recruited with a target sample size of 121 participants.

### Rejection induction

The experience of social rejection was manipulated using a German version of the future alone paradigm (FAP; 28]. Participants filled out personality questionnaires (i.e., RS, extraversion). The computer program automatically analyzed the extraversion score and provided participants with an accurate feedback of their responses, aiming to increase the plausibility of the “future life” feedback. Following this correct feedback, participants were provided with one of three randomly assigned feedbacks designed to manipulate their inclusion perception. The random allocation of participants to the three experimental groups (i.e., inclusions, medium rejection, severe rejection) ensured an equal number of participants in each group. Participants in the inclusion condition were told that they would have rewarding relationships throughout life. Participants in the medium intensity stress induction were told that they would lose approx. 71% of all of their friends and end up with 75% fewer relationships than the average individual. Participants allocated to the severe stress induction were told that they would end up completely alone later in life (for the specific feedback please refer to Bernstein and Claypool [28]).

### Social affiliation

**Fig 1:**
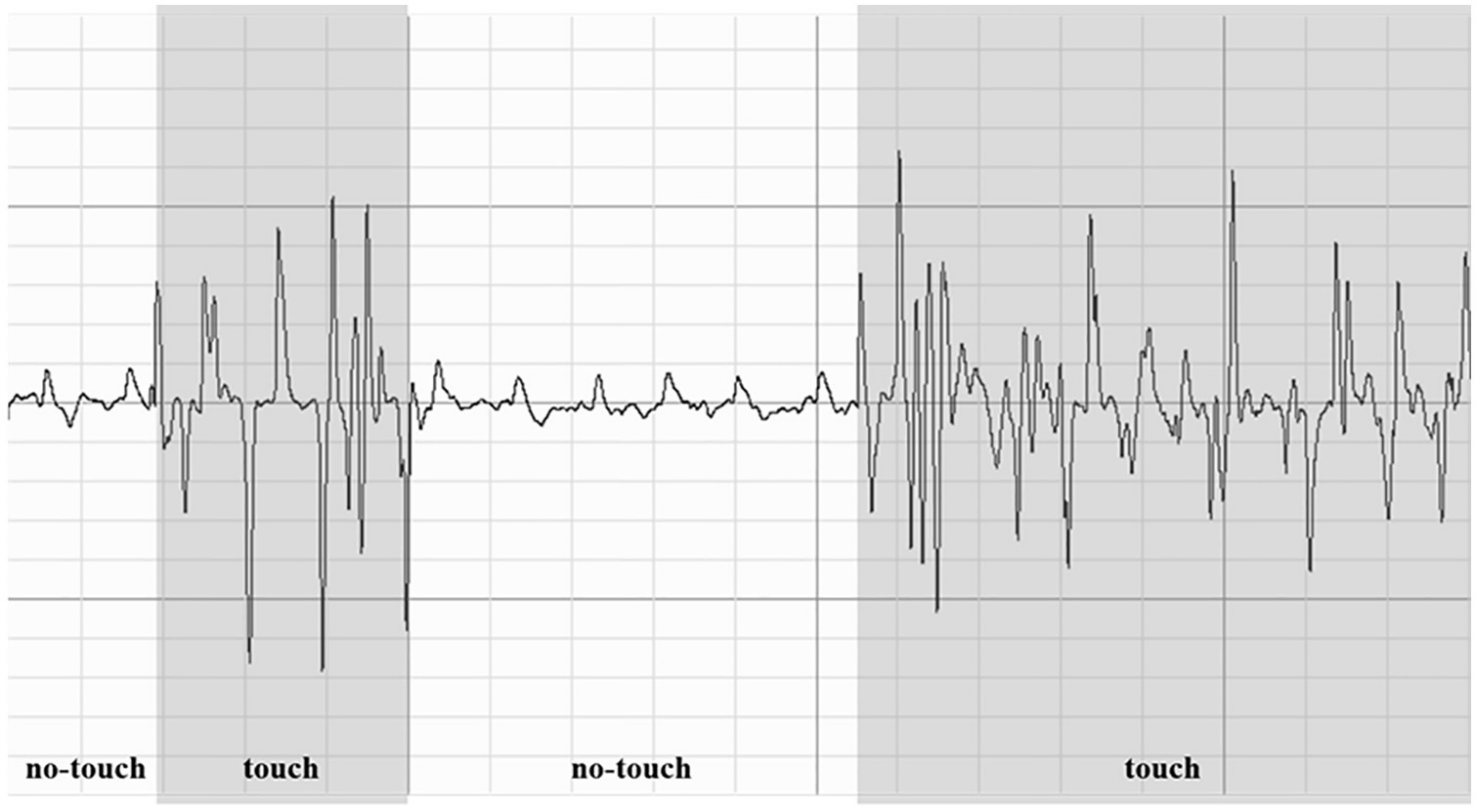
Illustration of a sample ICG signal of one participant during the social affiliation task. Electrical interference in the ICG signal due to touching of hands is highlighted in gray.

To assess social (re-)affiliative behavior, a task first introduced by Koslov [50] was implemented. At the beginning of the study, strip electrodes for impedance cardiography (ICG) were attached at the neck, below each clavicle, on the arcus costae and on the lower abdomen in the mid-clavicular line. The ICG signal was assessed with a sampling rate of 1 kHz and a low-pass filter for Z_0_ of 10 Hz. The applied AC frequency was 50 kHz. After obtaining verbal consent, a confederate, unknown to the participant, was invited to the room. The confederate was introduced as another participant and seated in a chair face-to-face with the participant. The confederate was wearing ICG electrodes and the pre-attached cables were connected to the Biopac system in the participant’s room. A table was positioned between the participant and the confederate and they were told to play for three minutes a touch-based communication game. The experimenter explained that the aim of this game was to investigate how people who cannot see or hear each other interact with one another. Therefore, a box was introduced on the table that had holes at both sides allowing the confederate and the participant to place their dominant hand in the box. The box was large enough to allow both to keep their hands hidden under the box without touching each other. The experimenter handed an international sign language (ISL) alphabet to both the participant and the confederate and explained that both were to communicate as many letters as possible to each other within a period of three minutes. For the seemingly random allocation of who was assigned to sign and who to guess the letters the experimenter held a box between the participant and the confederate and asked them to choose, who of them wanted to draw their role in the game. The confederate always offered the participant to draw, which was accepted by all of them. The participant was always assigned to the role of the guesser and therefore had to touch the hand of the confederate to determine the signed letter. Once they guessed the correct letter, the confederate changed to another letter. All letters were—unknown to the participant—predetermined and the same for all participants. In addition, both the confederate and the participant were instructed not to talk during the task. Since both were connected to an ICG module within the same amplifier system (Biopac MP150), the touching of hands caused a significant electrical interference in the ICG signal that clearly differed from any movement artifacts (see Fig 1). The total time of signal interference as scored in Acqknowlege 4.2, therefore, reflected the total time that the participants touched the confederate and was used as primary dependent variable. In addition, the number of correctly communicated letters was used as an indicator of task performance.

### Psychological data

#### Emotional reactivity

Changes in affect due to the induction of future rejection were assessed using the German version of the positive affect and negative affect scale (PANAS; [51,52]). The PANAS consists of 20-items questionnaire measuring positive (α = .81 − α = .88) and negative (α = .73- α = .88) affect state. Participants were asked to rate their current emotional state with the help of 10 positive (e.g. interested) and 10 negative (e.g. ashamed) adjectives on a Likert scale ranging from 1 (= not at all) to 5 (= extremely). The PANAS has been shown to have excellent psychometric properties [52].

#### Personality questionnaires

RS was measured using the short version of the Rejection Sensitivity Questionnaire [53] (α = .72). The scale consists of 9 scenarios such as “You approach a close friend to talk after doing or saying something that seriously upset him/her” and participants indicate on a 4-item scale how concerned they would be in that situation and if they think they would be accepted (very unlikely–very likely). The psychometric properties of the short version of the RSQ have been shown to be sufficient [53].

#### Extraversion

Extraversion was measured using the German version of the extraversion personality questionnaire (EPI; [54], α = .68). The questionnaire consists of 24 questions concerning social behavior and feelings (e.g., “Do you like mixing with other people?”). Participants were asked to agree or disagree with the personality questions asked. The scale has been shown to have good psychometric properties in previous studies [54,55].

### Procedure

**Fig 2:**
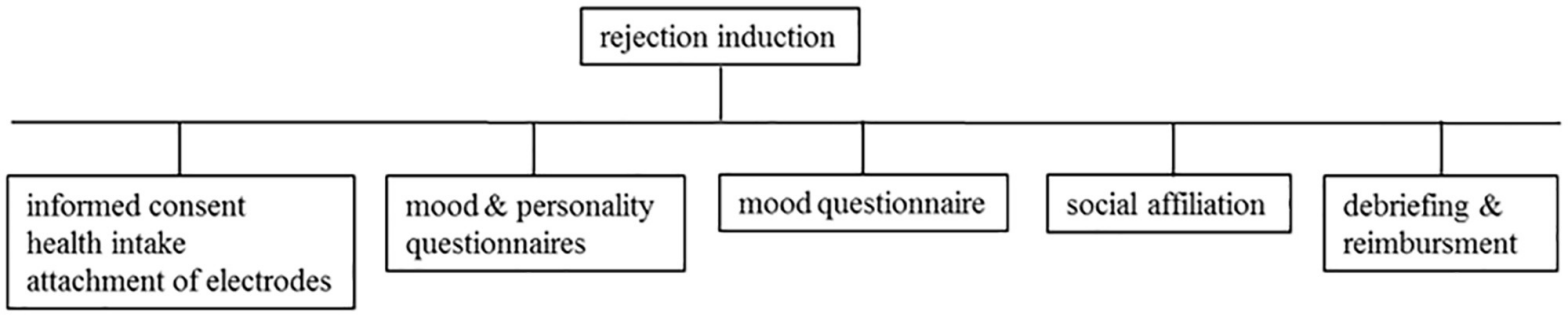
Illustration of the procedure of the study.

A female experimenter was always present during the session for health and safety regulations, sitting in a cubicle outside the visual field of the participant. After having signed the informed consent, participants were attached to the physiological equipment (impedance electrodes) and completed the PANAS and the personality questionnaires (see Fig 2). They then read through the FAP, followed by the PANAS. Participants were then asked if they were willing to interact with another participant. All participants agreed to the interaction. After verbal agreement, their social affiliative behavior was assessed using the Sign Language Task. Participants were debriefed at the end of the experimental session with regard to the personality feedback and were offered to contact the first author in case of further questions. All participants received a financial compensation of 20 Euros.

### Statistical analysis

All data were scored and analyzed using *AcqKnowledge 4*.*2* and *SPSS 21*. Kolmogorow-Smirnow and Mauchly’s tests were performed to test for the normal distribution and sphericity assumptions, respectively. Effect sizes are reported for any significant interaction or main effect using *Cohen’s d* statistic (for *t*-tests) or partial eta-squared statistics (η_p_^2^; for AN[C]OVA results). By convention, effect sizes of *d* = .20/ η_p_^2^ = .01, *d* = .50/ η_p_^2^ = .06, and *d* = .80/ η_p_^2^ = .14 reflect small, medium, and large effects sizes, respectively. Significance level was set to *p* < .05. In the case of significant Levene-test results, *t*- and *F*-values for unequal variances are reported.

Hypothesis Ia und III were analyzed together in a regression model. Two dummy variables were calculated: For the first dummy variable, participants who received the medium intensity rejection condition received the code 1 while participants from the inclusion and the severe rejection condition received the code 0. For the second dummy variable, participants in the severe rejection condition received the code 1 and all others the code 0. In case of significant results for hypothesis I, RS and its interactions with the two dummy variables were entered into the model. Interactions were dropped from the model in case of non-significance.

Hypotheses Ib and HIV were analyzed in a second regression model using total amount of correctly identified letters as dependent variable and the two dummy variables from regression 1 (and then from the regression 2), RS as well as their interactions as predictors. Again, non-significant interactions were excluded from the final model.

Hypothesis II was analyzed using repeated-measures analysis of variance (ANOVA) for negative affect (NA) and positive affect (PA), respectively, as dependent variable across both time points (at baseline and after the rejection manipulation). The specific FAP condition (inclusion, medium rejection, severe rejection) was used as between- subject variable. Post-hoc analyses using ANOVAs and student t-tests were carried out in case of significant interactions between time and condition.

An adjusted Bonferroni correction (α/n-1) was used for all post-hoc (t-test) analyses to avoid type I error inflation. For three post-hoc analyses p-values had to be smaller than .025 to be considered significant.

## Results

### Hypotheses Ia and III

The regression model was significant (*F*(3,116) = 4.466, *p* = .005, *R*^2^ = .11; Table 1). Medium intensity rejection significantly predicted total time of affiliative behavior, as did RS. Severe social rejection, however, did not predict the duration of affiliative behavior. This suggests that participants who experienced an inclusion scenario touched their partners’ hands as long as participants who were severely rejected, whereas medium rejected participants sought more physical contact that included participants. Higher scores in RS were associated with shorter hand touching durations. Inclusion of the interaction effects between RS and the experimental conditions (RS*dummy 1: β = -0.32, *p* = .915, CI[-4.494; 4.033]; RS*dummy2: β = -.050, *p* = .879, CI[-4.654; 3.989]) did not result in improved model fit (change in *R*^2^ < .001, *p* = .988).

**Table 1:** Results of the first regression analysis for hypothesis III. Dependent variable: predicted total time of affiliative behavior.

	beta	95% confidence interval [LLCI; ULCI]	p-value
Medium intensity rejection	β = .255	[3.590; 32.047]	*p* = .015
Severe intensity rejection	β = .137	[-4.672; 23.842]	*p* = .186
Rejection sensitivity	β = -.231	[-3.999; -.528]	*p* = .011

*Note*. LLCI = lower limit 95% confidence interval; UCLI = upper limit 95% confidence interval. *N* = 121.

### Hypotheses Ib and IV

The regression model was not significant (*F*(3,97) = 1.953, *p* = .126, *R*^2^ = .057; Table 2). Inclusion of the interaction effects between RS and the experimental conditions (RS*dummy 1: β = .179, *p* = .581, CI[-.376; .666]; RS*dummy2: β = .616, *p* = .077, CI[-.052; .989]) did not result in improved model fit (change in *R*^2^ = .032, *p* = .196).

**Table 2:** Results of the regression analysis for hypothesis IV. Dependent variable: task performance.

	Beta	95% confidence interval [LLCI; ULCI]	p-value
Medium intensity rejection	β = -.250	[-3.782; -.199]	*p* = .030
Severe intensity rejection	β = -.099	[2.606; 1–017]	*p* = .386
Rejection sensitivity	β = -.090	[-.116; .314]	*p* = .364

*Note*. LLCI = lower limit 95% confidence interval; UCLI = upper limit 95% confidence interval; *N* = 121

### Hypothesis II

**Fig 3:**
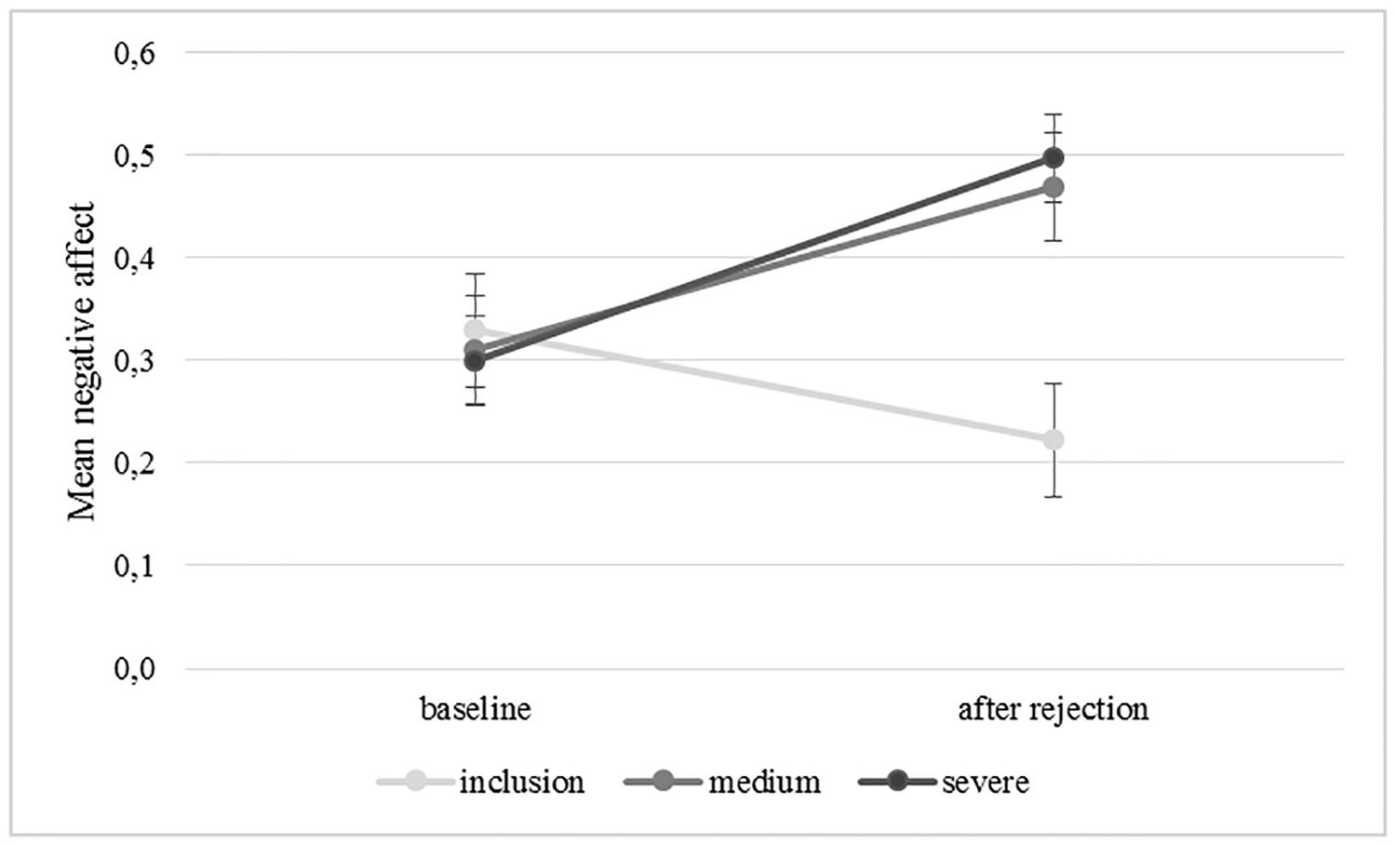
Negative affect at baseline and after rejection. Error bars indicate one standard error. *N* = 121. Likert scale ranging for the negative affect from 1 (= not at all) to 5 (= extremely).

Negative affect increased significantly over time (*F*(1,117) = 4.772, *p* = .031, η_p_^2^ = .039; CI[-.159, -.008]; see Fig 3). The interaction between time and condition (inclusion, medium intensity rejection, severe rejection) was significant (*F*(2,117) = 6.336, *p* = .002, η_p_^2^ = .098). The main effect for condition was not significant (*F*(2,117) = 1.677, *p* = .191, η_p_^2^ = .028). Post-hoc t-tests revealed a significant decrease in negative mood over time in the inclusion group (*t*(39) = 3.107, *p* = .004, *d* = .345, CI[0.038; 0.177]). Negative affect increased non-significantly over time in the medium intense (*t*(39) = -2.009, *p* = .052, *d* = 0.353, CI[-.321; .001]) but significantly in the severe rejection condition (*t*(39) = -2.649, *p* = .012, *d* = 0.505, CI[-.348;-.047]). To check for group differences at baseline and after the manipulation, two post-hoc ANOVAs were calculated with negative affect at the respective time points and condition (inclusion, medium, severe) as between-subject factor. There were no differences in negative affect at baseline (*F*(1,117) = .091, *p* = .913, η_p_^2^ = .002). Groups, however, differed significantly with regard to negative affect after the feedback (*F*(1,117) = 4.421, *p* = .014, η_p_^2^ = .070), with both rejection conditions scoring significantly higher on negative affect directly after the manipulation than the inclusion condition (medium rejection: (*t*(78) = 2.510, *p* = .015, *d* = 0.561; CI[.099; .050]), severe rejection: (*t*(78) = 3.100, *p* = .003, *d* = 0.693, CI[.452; .098]). Individuals in the medium and severe rejection conditions did not differ with regard to self-reported negative affect after the feedback (*t*(1,78) = -.231, *p* = .814, *d* = .003; CI[.-.259; .204]).

**Fig 4:**
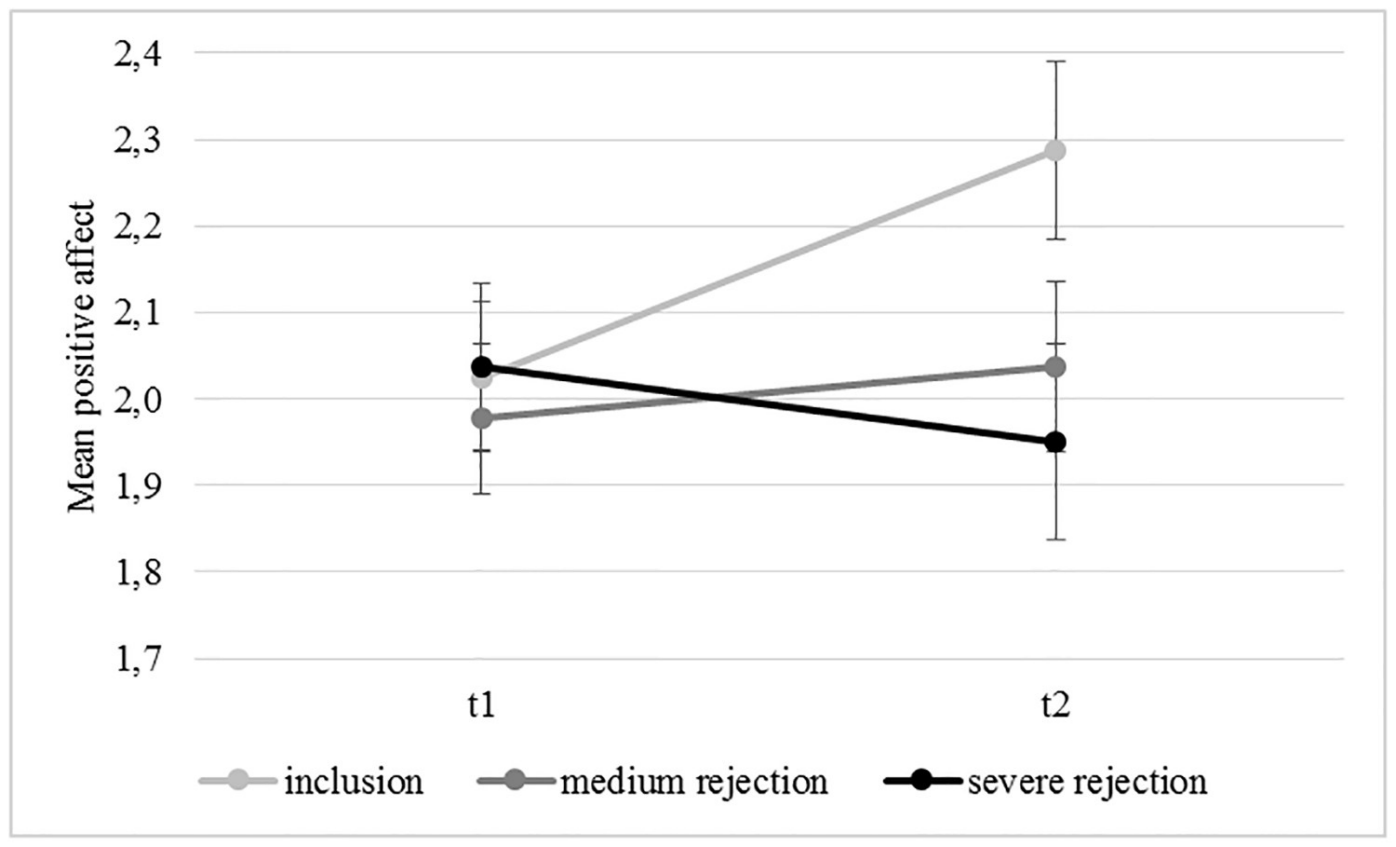
Positive affect at baseline and after rejection. Error bars indicate one standard error. *N* = 121. Likert scale ranging for the positive affect from 1 (= not at all) to 5 (= extremely).

While the main effect for time was not significant for positive affect (*F*(1,117) = 3.034, *p* = .084, η_p_^2^ = .025; see Fig 4), the interaction between time and condition was significant (*F*(2, 117) = 5.089, *p* = .008, η_p_^*2*^ = .080). The main effect for condition was not significant (*F*(2,117) = 1.001, *p* = .371, η_p_^2^ = .017). Post-hoc t-tests revealed a significant increase in positive mood over time in the inclusion group (*t*(39) = 3.798, *p* < .001, *d* = .51, CI[-.402,-.123]). There was no change in positive affect in the medium intensity rejection group (*t*(39) = -.739, *p* = .464, *d* = .104, CI[-.224;.104]) and in the severe rejection group (*t*(39) = 1.058, *p* = .296, *d* = .131, CI[-.079; .254]). To check for group differences at baseline and after the manipulation, two post-hoc ANOVAs were calculated with positive affect at the respective time points and condition (inclusion, medium, severe) as between-subject factor. There were no differences in positive affect at baseline (*F*(1,117) = .122, *p* = .885, η_p_^2^ = .002). Groups only differed at trend level with regard to positive affect after the feedback (*F*(1,117) = 2.779, *p* = .066, η_p_^2^ = .066), with both rejection conditions scoring lower on positive affect directly after the manipulation than the inclusion condition (medium rejection: (*t*(78) = 1.754, *p* = .083, *d* = .392, CI[-.038; .534), severe rejection: (*t*(78) = 2.204, *p* = .030, *d* = .495, CI[.033; .643]).

## Discussion

Threats to the social self adversely affect psychological well-being and physical health [2, 3, 8, 11,12, 56]. Perceived social rejection is related to trait RS, i.e. a personality trait characterized by chronic attentional focus on and biased interpretation of socially threatening cues, and/or to situational circumstances. The present study is–to our knowledge–the first to investigate how anticipated rejection alters physical interactions with an unknown person. RS can motivate the individual to protect oneself from threatening social situations [45,46]. We tested the hypothesis that RS is negatively related to affiliative behavior. Previous findings suggest an U-shaped response relationship between the intensity of the experience of social rejection and emotional pain, with medium intensity social rejection leading to increases in negative affect and physical sensations, and severe social rejection resulting in emotional withdrawal and physical numbing [28]. In the present study we investigated this hypothesis with regard to social (re-) affiliation after rejection with the notion that medium social rejection motivates the individual to ‘repair’ the perceived damage to the social self by seeking social contact [28]. Severe social rejection, however, was expected to elicit a threat response that results in emotional and social withdrawal [28].

Participants who were excluded perceived more negative affect after the manipulation compared to those in the inclusion condition, supporting previous findings [15,28,57]. There were no differences with regard to positive affect, however, which is consistent with previous findings [57]. Contrary to our hypotheses, there was no evidence for emotional numbing in participants who were severely rejected. This contrasts with findings by Bernstein and Claypool [28], who found severe social stress to result in emotional numbing.

Although we did not find evidence for the hypothesized differences in self-reported mood between the medium and severe rejection groups, we next examined behavioral differences with regard to contact seeking. With regard to (re-) affiliation after rejection, there were indeed differences between the medium intensity and inclusion conditions with participants in the medium intensity social rejection condition seeking more physical contact, while participants in the severe rejection condition touched the confederate as long as included participants. The current findings, therefore, support the notion of the reconnection hypothesis that medium intensity rejection leads to increased affiliation behavior with others [28,57]. Participants in the severe rejection condition might have been concerned with protecting themselves from further harm, which might be, in this study, an anticipated evaluation by the confederate or the experimenter due to low task performance or excessive touching. The most adequate and, therefore, safest social behavior in their perspective might have been focused, and purely goal oriented physical contact initiation. This might explain why there was no evidence for social withdrawal in severely rejected participants [28].

Social context substantially influences whether people seek or avoid social closeness [58]. Characteristics of the social interaction task might, therefore, affect its ability to induce social withdrawal. Physical contact is one of the most powerful sensory experiences [59]. Physical touch between family members or strangers has been shown to transmit discrete emotions [60–62] and to promote stress contagion [59]. While participants in the medium intensity rejection condition were willing to communicate with their partners by seeking social closeness, included participants, who did not differ in their affiliative behavior from severely rejected participants, kept more to themselves. This might be either because they did not need social comfort that much or because they were trying to avoid it and its consequences as much as socially possible. Alternatively (or additionally) touching a strangers hand may be perceived as uncomfortable as it creates a level of physical intimacy that would normally only occur between friends or intimate partners. Physical contact might, therefore, have been reduced to the minimum needed for successful task completion. If social withdrawal was an evoked behavioral tendency in the severe rejection condition, participants in the inclusion condition might also have tried to reduce contact to a minimum, which would explain why we did not find differences between the inclusion and severe rejection conditions. Future studies should investigate if changes in the paradigm by touching friends or intimate partners [63] could reduce awkwardness of the situation and would lead to different behavior outcomes in the inclusion or severely rejected participant groups.

The interaction between RS and stress intensity on physical touch duration was not significant. This was unexpected, as one would assume that people high in RS perceive the intensity of the manipulation stronger and, therefore, show a more extreme reaction. Alternatively, individuals high in RS may be used to the feeling of being rejected and may, therefore, not be as surprised about the future alone feedback than individuals low in RS. Nevertheless, there was no interaction between RS and condition. Many personality variables don’t moderate reactions to social rejection (e.g., self-esteem, social anxiety) [64–66]. Hence, it may simply be that for our outcome variable, RS didn’t overcome the strong effects of rejection. While RS did not moderate effects of rejection in this study, there are studies, where RS could be identified as important moderator [67, 68]. Future studies should therefore include the assessment of RS while investigating the effects of state rejection and analyze trait RS as potential moderator.

Importantly, while individuals who experienced medium intensity social rejection touched their partners more, they seemed to have had more difficulties in identifying the signed letters as indicated by a trend in the statistical analysis. Performance during the cooperative interaction may have competed with the goal of seeking affiliation in such a way that the latter took preference over the given task to identify letters. Likewise, participants may have spent more time exploring the sign to be more certain about their guesses. Although this finding reached only trend level, it reflects the importance of investigating the relationship between rejection intensity, social affiliation and performance further, as this would be of importance for occupational psychologists.

RS was negatively correlated with duration of physical touch. This supports previous findings showing that people high in RS might forestall rejection and prevent uncomfortable closeness in situations in which social withdrawal is possible [45, 46]. The tasks made social withdrawal possible and legitimate to a certain point (i.e., given the identification of some letters), as both hands were hidden in the box. Because the task was formulated to be goal oriented (i.e., to identify letters), and was also unfamiliar, there would also have not been any previously known social norm dictating how long one normally touches a strangers’ hand, thus decreasing potential barriers to social withdrawal. Hence, this is the first study to show that RS affects social interaction with strangers, even under non-threatening circumstances that do not imply personally meaningful or long-term contacts. It appears that rejection sensitivity impacts social behavior even in goal-oriented social tasks where personal protection is not necessary. These results are important for the understanding of the mechanisms explaining why people high in RS have more difficulties in building a social network compared to individuals low in this trait. Already at the initiation level, highly rejection sensitive individuals show signs of social avoidance that might render it difficult for them to connect and build strong relationships. This strategy is ultimately self-defeating as the withdrawal from social interactions fosters the likelihood of experiencing loneliness and thereby increasing sensitivity to rejection [69]. Given the important relationship between RS, loneliness and well-being [39, 42, 46], it seems crucial to focus onto the process of initiating social contacts in interventions designed to support those high in RS. RS could be reduced by increasing social efficacy and social self-esteem, thereby increasing the probability of being liked and integrated by others [70]. Additionally, individuals high in RS could be trained to seek for alternative interpretations of the behavior of others, when becoming aware of their feeling of being rejected [69]. Walton and Cohen [3] implemented a social-belonging intervention designed to reduce perceptions of threat in a group of stigmatized minority students by framing adversity as common and transient. The intervention led to long-term increases in well-being, health and academic performance. It might be promising to adapt this intervention to RS by framing rejection as common and transient and then examining if rejection sensitive individuals show improvements in well-being and social approach as a result. The ISL-task used in this study could be used as objective outcome measure for such interventions as well as for the therapy of depression and social anxiety [69, 71]. It would be important for the development of more specific interventions to address whether individuals high in RS are aware of their reduced contact seeking.

### Limitations

Limitations of the present study concern the sample, which was restricted to a female student population, which restricts the generalizability of the results to the population at large. Nevertheless, the current study was appropriately powered, so the results offer support to warrant replication in a mixed-gender sample to investigate potential sex-differences in these effects. Previous studies have not found gender differences with regard to RS [39, 72]. Nevertheless, gender differences exist with regard to the consequences of RS, in as much as in women RS is more strongly associated with pessimism than in men [73]. It remains unclear, therefore, whether men would also have shown a negative correlation between RS and the duration of physical touch as was observed in women. It seems important, therefore, to replicate the RS–physical touch relationship in a male sample.

The current study shows that medium rejected participants touched their partner longer than included participants. While the motivation to refrain from social contact for participants under severe rejection due to fear of further rejection for instance seems clear, we have less insight as to why included participants might limit their social contact initiation. It is possible that both behaviors are about motivation to connect [74]. One could argue that severely rejected participants are particularly motivated to avoid further rejection and therefore avoid contact to ensure they don’t get additionally hurt, while included people are less motivated to have contact simply because their sense of belonging was not threatened. The exact underlying mechanisms of contact initiation remain therefore unclear and warrant further investigations.

As we did not find support for the hypothesized U-shaped relationship between the intensity of rejection and negative affect, replication of the findings are necessary. Since this is–to our knowledge- the first study in a German sample, it is difficult to disentangle whether the here found results are due to the intervention or reflect a true effect. As the severe social rejection condition did not result in emotional numbing as measured with the PANAS, it might be that the PANAS is not an appropriate measure to display the emotional response as elicited by the FAP. Other measures such as the need threat scale, for instance, might be worthwhile testing in future studies to reflect emotional changes as response to the FAP. There is also a risk that the condition was not efficient enough. This might be due to cultural differences between the US and a German speaking population. Rejection inductions such as the non-verbal Cyberball game (e.g., [75]) might have had advantages over the FAP. Nevertheless, the FAP paradigm has been shown to elicit different intensities of rejection (inclusion, medium and severe rejection), and as we hypothesized that rejection sensitivity would show a differential effect on affiliative behavior depending on rejection intensity, we chosen the FAP paradigm for this study. Rejection as elicited by the Cyberball game has been shown to induce rejection of medium intensity as compared to the FAP [28]. The Cyberball game could be used to replicate the finding of increased affiliative behavior under medium rejection as compared to inclusion. Future studies should, therefore, develop culturally adapted versions of the FAP. Nevertheless, as similar mood responses were observed in the medium and severe rejection conditions, the manipulation seems to have worked but lead to different behavioral responses. The ISL-task as realized in this study only allows for the analysis of the duration of touch. Future studies could look into possibilities to measure the quality/intensity of touch as well for instance by using infrared cameras.

### Conclusion

This study is the first to show that individuals scoring high in RS seek less physical contact with strangers than those low in RS. The current results support the notion by Bernstein and Claypool [28] that medium intensity social stress leads to social (re-) affiliation. While there was neither evidence for emotional withdrawal nor for changes in performance in the severely rejected group, medium intense rejection resulted in the expected behaviors. Although participants in the medium intense rejection group had more opportunity to identify the letters as they touched the hand of their partner for longer, they performed worse in identifying the letters compared to included or severely rejected participants. This result has important implications for occupational psychologists by offering a framework to better understand performance attrition in the context of bullying and harassment.
